# Supplementary figures and images for: S-farnesylation is essential for antiviral activity of the long ZAP isoform against RNA viruses with diverse replication strategies
Source: PLoS Pathog. 2021 Oct 25;17(10):e1009726. doi: 10.1371/journal.ppat.1009726 (PMC8568172; doi:10.1371/journal.ppat.1009726)

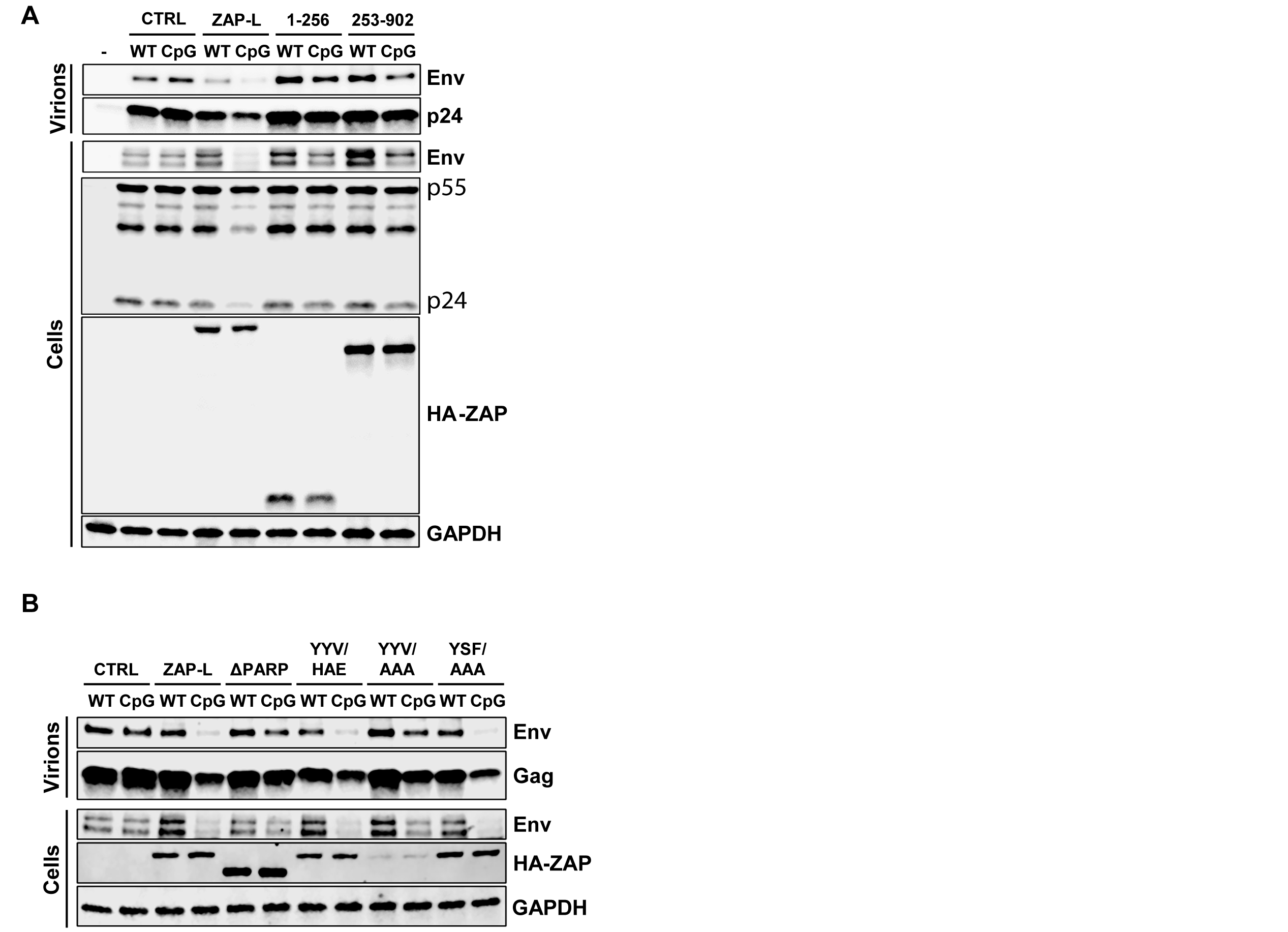

Supplement: S1 Fig — Representative western blots of the experiments shown in (A) Fig 1B and (B) Fig 2D. (TIF) [file ppat.1009726.s001.tif]

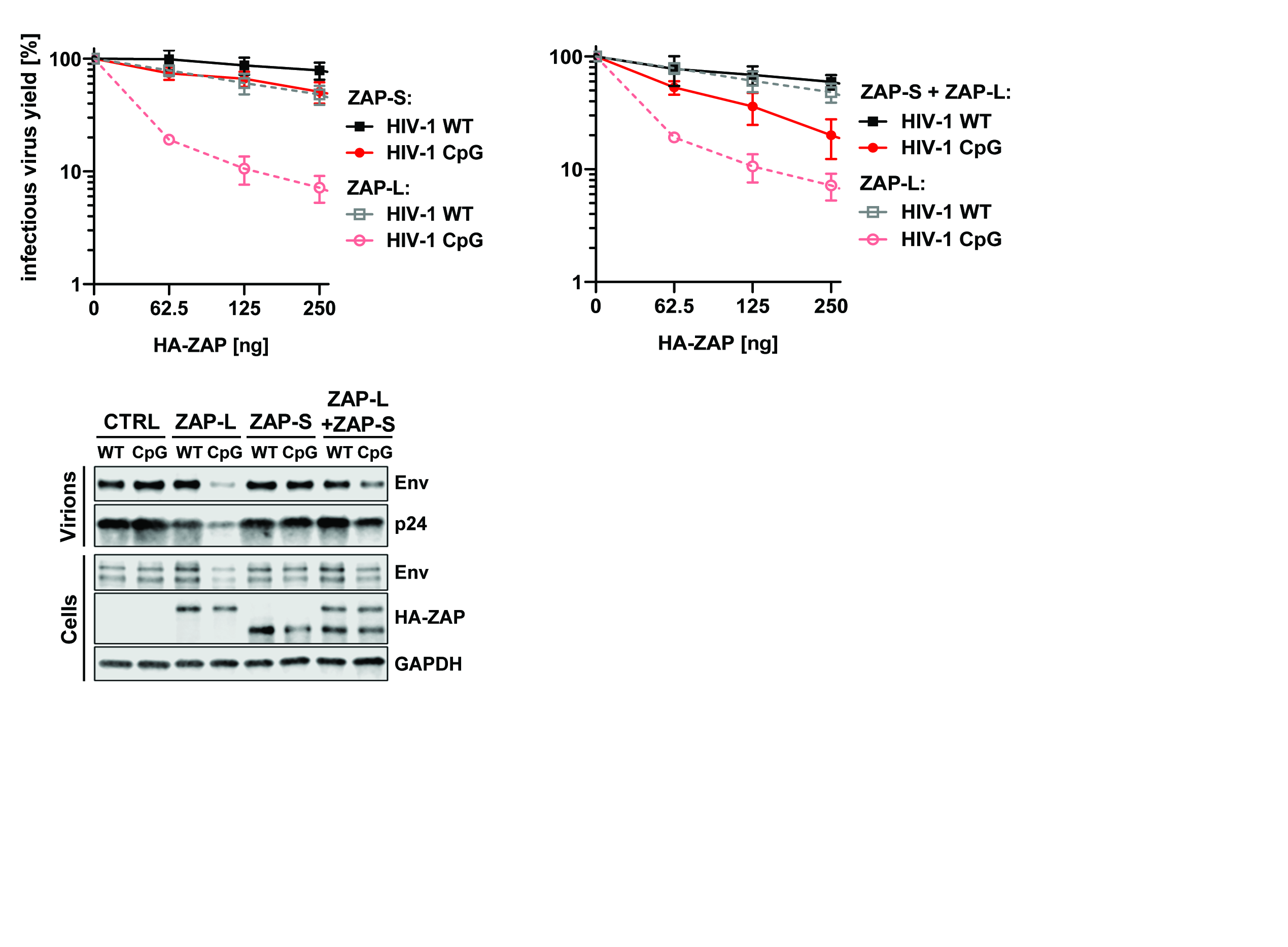

Supplement: S2 Fig — Infectious virus yield from HEK293T ZAP KO cells co-transfected with wild type (WT; black) HIV-1 and CpG-enriched mutant (CpG-high; red) viruses and increasing doses of pcDNA HA-ZAP constructs encoding ZAP-L (dashed lines), ZAP-S or 1:1 ratio of both isoforms up to 250ng each (solid lines). Values were normalized to infectivity in the absence of ZAP for each virus (100%). Mean of n = 5 +/- SD. Lower panel: representative western blot (250ng HA-ZAP). (TIF) [file ppat.1009726.s002.tif]

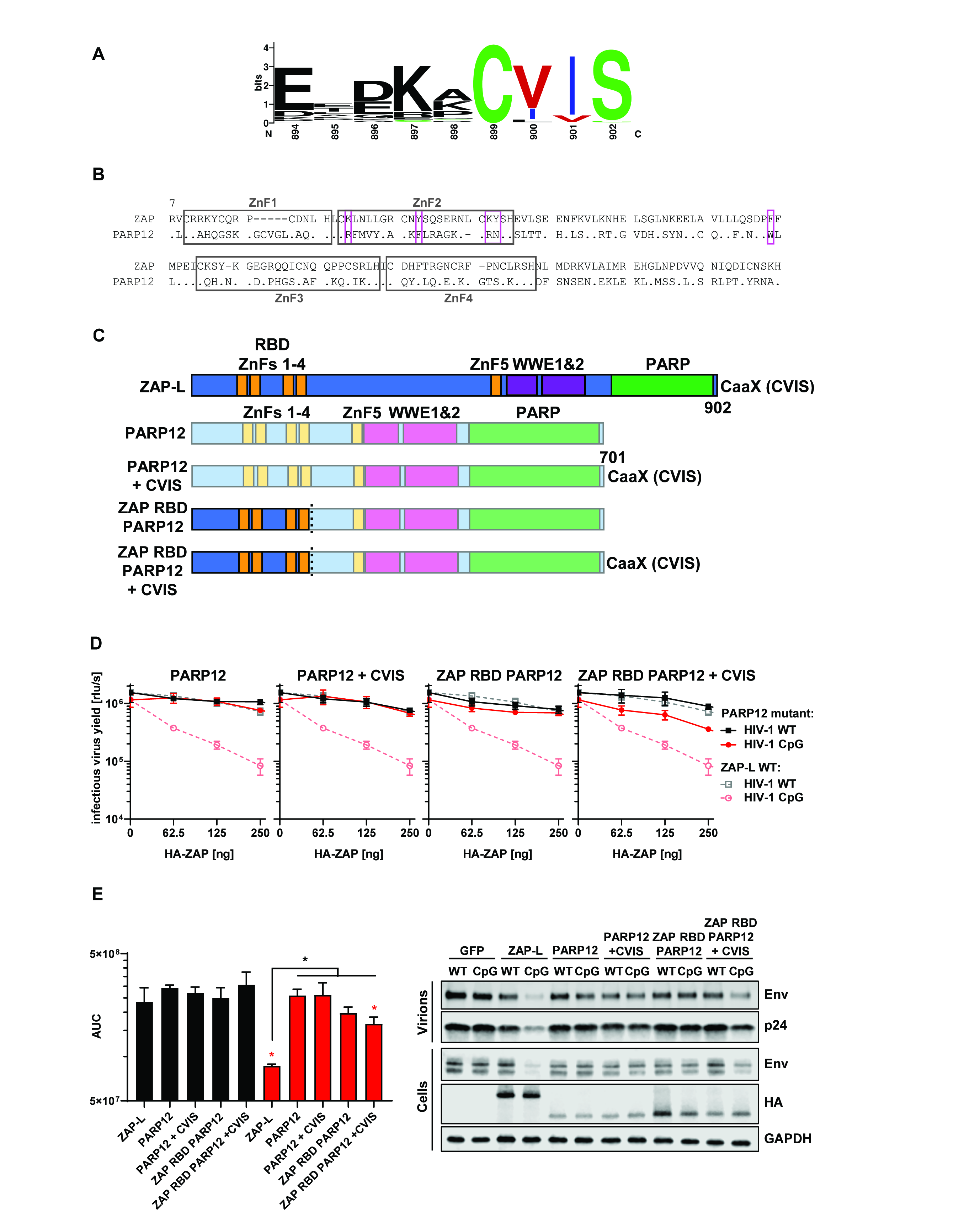

Supplement: S3 Fig — (A) Logo plot of C-termini of mammalian and bird ZAP-L orthologues from NCBI database. Highly conserved serine 901 determines targeting by cellular farnesyl transferase which prenylates highly conserved cysteine 899. (B) Alignment of RNA-binding domains of human ZAP and its paralogue PARP12. Four zinc fingers (grey boxes) and ZAP residues interacting with CpG dinucleotide in bound RNA (highlighted in pink) are indicated. (C) Schematic showing the domain organisation of ZAP-L, PARP12 and PARP12/ZAP chimeric constructs. (D) Infectious virus yield from HEK293T ZAP KO co-transfected with WT (black) and mutant (red) virus and increasing concentration of pcDNA HA-ZAP-L CTRL (dashed line), PARP12, or ZAP/PARP12 chimera (solid lines). (E) corresponding AUC values and representative western blot (250ng). Mean of n = 3+/- SD. * p<0.05 for HIV-1 CpG compared to HIV-1 WT for the same ZAP construct. * p < 0.05 for the comparisons demarked by the lines. (TIF) [file ppat.1009726.s003.tif]

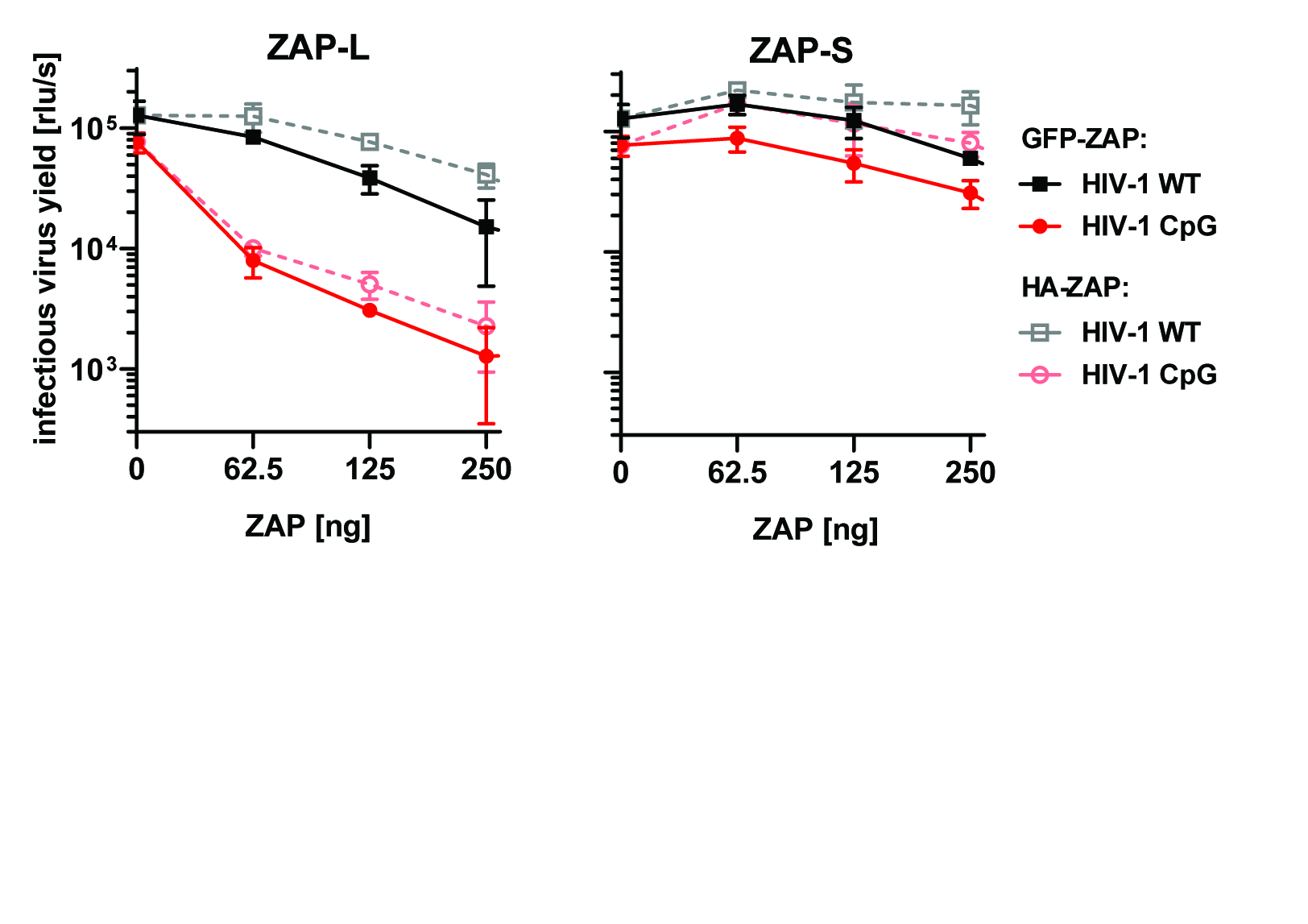

Supplement: S4 Fig — Infectious virus yield from HEK293T ZAP KO cells co-transfected with wild type (WT; black) HIV-1 and CpG-enriched mutant (CpG-high; red) viruses and increasing doses of pcDNA ZAP with N-terminal hemagglutinin tag (HA) or monomeric enhanced green fluorescent protein (GFP) tag. Mean of n = 3 +/- SD. (TIF) [file ppat.1009726.s004.tif]

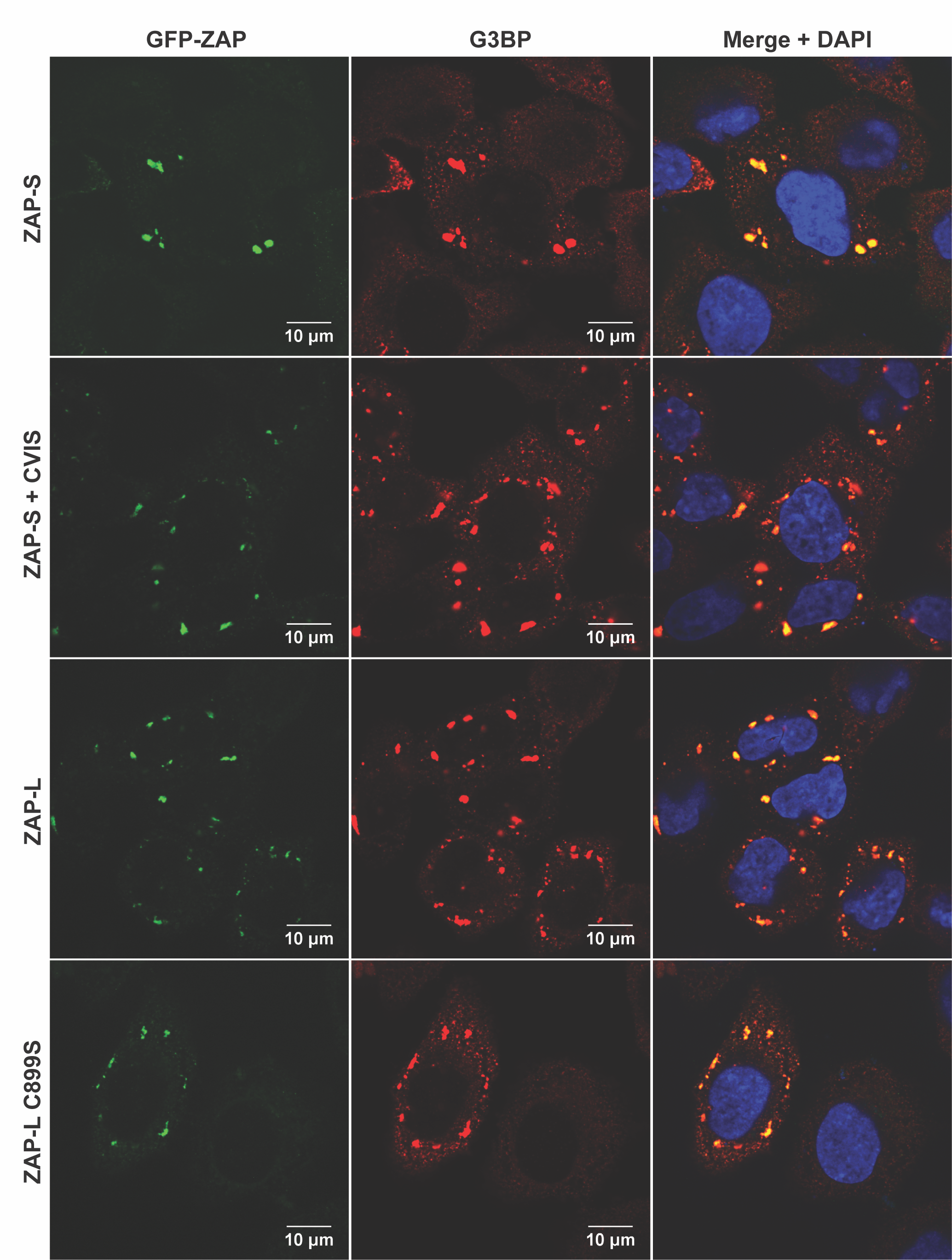

Supplement: S5 Fig — HeLa ZAP KO cells were transfected with 125ng GFP-ZAP (green) and stained for stress-granule marker G3BP (red) following treatment with 100ng of poly(I:C). DAPI staining shows cell nuclei (blue). (TIF) [file ppat.1009726.s005.tif]

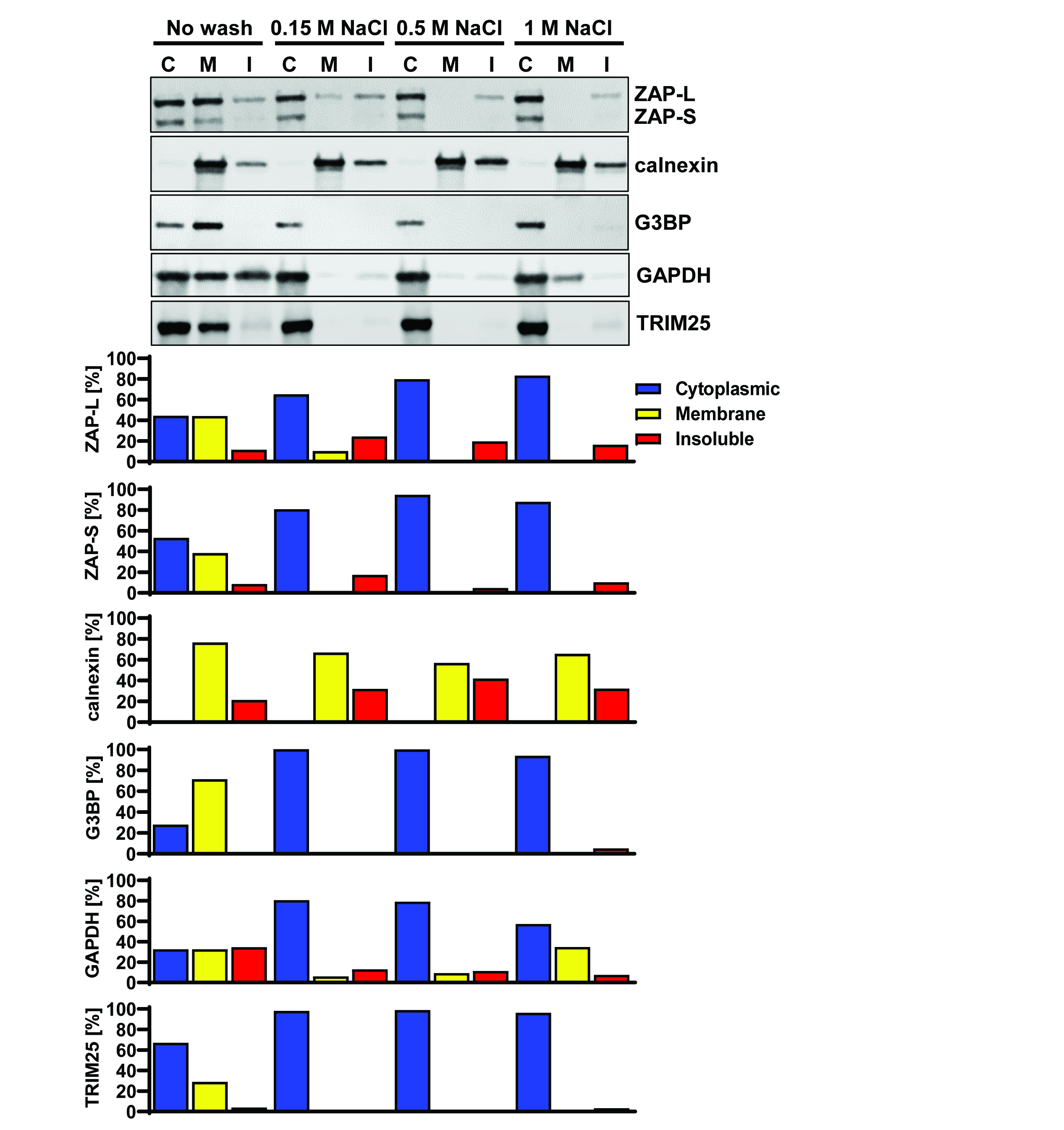

Supplement: S6 Fig — Western blot and protein quantification following fractionation of HEK293T cells. Cytoplasmic (C), membrane (M) and insoluble (I) fractions are shown, with relative levels of endogenous ZAP-L and ZAP-S, as well as controls calnexin (membrane fraction control), and G3BP, GAPDH and TRIM25 (cytoplasmic fraction controls). (TIF) [file ppat.1009726.s006.tif]

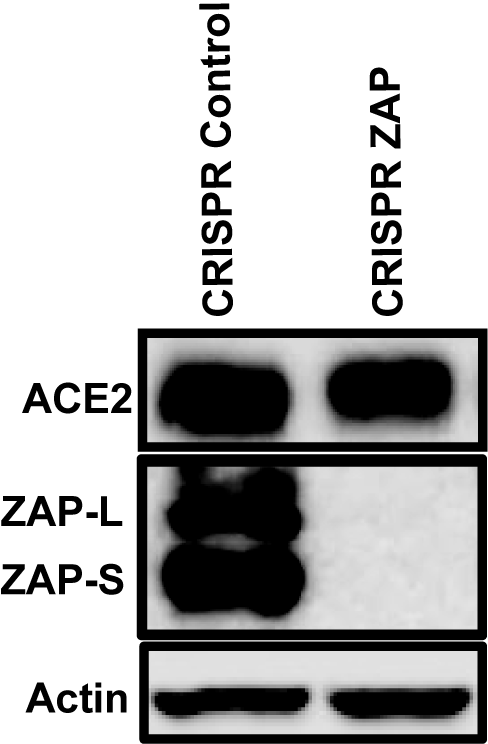

Supplement: S7 Fig — Western blot of CRISPR control and ZAP CRISPR A549-ACE2 cells demonstrating that ZAP has been knocked out in the ZAP CRISPR cells. (TIF) [file ppat.1009726.s007.tif]
