# Supplementary material for: S-farnesylation is essential for antiviral activity of the long ZAP isoform against RNA viruses with diverse replication strategies
Source: PLoS Pathog. 2021 Oct 25;17(10):e1009726. doi: 10.1371/journal.ppat.1009726 (PMC8568172; doi:10.1371/journal.ppat.1009726)
Supplement: Attachment — Submitted filename: Kmiec et al Response to reviewers_V2.docx [file ppat.1009726.s008.docx]

We thank the reviewers for their insightful comments that have improved this manuscript. To address their comments, we have added new data and rewritten the text to make it more clear and concise.

**Reviewer's Responses to Questions**

**Part I - Summary
Please use this section to discuss strengths/weaknesses of study, novelty/significance, general execution and scholarship.**

**Reviewer #1: Kmiec and colleagues show that the catalytically inactive poly-ADP-ribose polymerase (PARP) domain of the long ZAP isoform (ZAP-L) is essential for CpG-specific restriction of HIV-1 and SARS-CoV-2. Their results clarify the determinants outside the CpG RNA-binding domain of ZAP that are important for antiviral activity and highlight the role of S-farnesylation and membrane association in ZAP-mediated antiviral function. Overall, the experiments were well designed and performed and the main conclusion is supported by the results. The findings are important for better understanding the mechanisms of ZAP-mediated viral restriction. However, there are some limitations should be addressed (see below comments in details).**

**Reviewer #2: Kmiec et al show that the CaaX signal for farnyslation of ZAP-L is important for its antiviral activity for CG-rich HIV-1 and SARS-CoV-2. Importantly, they are able to confer this increased antiviral activity on ZAP-S by appending this signal. The mutants also correlated with increased affinity to ZAP co-factors KHNYN and TRIM25 and cellular localization to internal membranes. This is an impressive amount of work and is convincing. My major comments are in regards to the manuscript organization and clarification of what is new versus what is confirmation.**

**Reviewer #3: This paper reports interesting findings concerning the antiviral activity of ZAP, which targets viral RNAs. The major conclusion is that the C-terminal PARP domain (an enzymatically inactive one) contributes significantly to the activity, especially on the major target studied here, CpG-rich HIV viral RNAs. In addition, mutations affecting farnesylation also impact activity, likely by preventing correct intracellular localization to vesicular membranes. Both PARP domain and farnesylation are needed for normal interaction with cofactors TRIM25 and KHNYN. These findings are interesting and the data are solid though the presentation is hard to parse. I found many of the interpretations of the data to be unclear – I didn’t know what to make of the data in many experiments. I think the paper needs to focus on the more clear-cut results, to state the conclusions more simply, and give the harder-to-interpret data less time or weight.

The literature on the importance of the PARP domain for ZAP activity seems very confused (as noted, line 170), and I think this paper will add to the confusion. ZAP-L seems generally to have more activity than ZAP-S, but the fold increase seems to vary widely in different papers, most likely because different targets are being assayed (and maybe because different cells are being used, with different levels of expression of endogenous ZAP, a good point). Residues in the PARP active site (the inactive site) seem essential sometimes and yet not in the case of ZAP-S. There would seem to be an opportunity here to sort this out, or at least voice an opinion.

Overall I think the language creates confusion as to when a ZAP construct is said to have “CpG-specific activity”. Do we mean it has no activity on a CpG-low target? Or that it has some basal activity on a CpG-low target and much higher activity on a CpG-high target? And when a construct is said to have “no CpG-specific activity”, presumably it means it has roughly equal activity on CpG-high and -low targets – but how much activity does it have on these two – is it high or low, compared with a construct that is “CpG-specific” acting on a CpG-high target?
Maybe we need to be talking about a measure of selectivity – like the ratios of activity on the two targets? Or maybe we need to establish some kind of scale, or a standard to compare to? Maybe pick a maximal setting, call it 100%, and then talk about % of that activity that is seen with a given construct and target? Whatever, the present description is very confusing. I think when describing the activity of any mutant, we should always be told its activity on a CpG-high and a CpG-low target. Period.**

**One example of what is unclear is epitomized by a sentence in the introduction: (lines 91 ff.): “we identified that the PARP domain and CaaX box found in ZAP-L, but not ZAP-S, are required for antiviral activity against CpG-enriched HIV-1 and SARS-CoV-2, explaining why ZAP-L is much more antiviral against these viruses than ZAP-S”. If these domains were “required” then ZAP-S would have no activity. But ZAP-L has “much more activity”, so ZAP-S apparently has some. Which is it? Presumably “required” is too strong. Or maybe the second half of the sentence should be deleted.

Maybe one solution is to just talk about activity on the chosen CpG-high HIV substrate and basically forget the wild-type CpG-low HIV, where activity is apparently low anyway. Or else do the CpG-high first through all the mutants, make all the important points, and then go back and consider the CpG-low separately, where not much can be said anyway.**

We apologise for the confusion created by our use of “CpG-specific activity”. We have addressed this two ways in this manuscript. For the HIV-1 experiments, we analyse the CpG-specific effect by comparing ZAP antiviral activity on the wild type and CpG-enriched virus. In addition, we have analysed the effect of mutating a CpG contact residue in ZAP, Y108, to alanine and analysing its antiviral activity on CpG-enriched HIV-1 and SARS-CoV-2. Since ZAP-L Y108A loses most of its antiviral activity for both viruses, we hypothesise that ZAP antiviral activity for both viruses requires it to bind CpGs. To avoid confusion, we have rewritten the text to make clear the CpG-specific effect and also removed this term from the title of the paper.

**Part II – Major Issues: Key Experiments Required for Acceptance
Please use this section to detail the key new experiments or modifications of existing experiments that should be absolutely required to validate study conclusions.

Generally, there should be no more than 3 such required experiments or major modifications for a "Major Revision" recommendation. If more than 3 experiments are necessary to validate the study conclusions, then you are encouraged to recommend "Reject".**

**Reviewer #1:**

**1. The whole study is dependent on ZAP KO HEK293T cells and transient transfection assays to evaluate antiviral effects of ZAP variants compared to WT proteins. It is important to know the levels of overexpressed ZAP compared to endogenous proteins in physiologically relevant cell types. For instance, CD4+ T cells for HIV-1 infection and airway epithelial cells SARS-CoV-2 infection. The authors can either perform some key experiments using these cell types or at least compare ZAP levels in transfected HEK293Twith these cell types.**

We understand the reviewer’s concern regarding ZAP levels. In fact, the first author of this manuscript, Dorota Kmiec, characterized this for her previous paper using ectopic expression of ZAP in HEK293T ZAP knockout cells (Kmiec et al 2020 mBio. 11:e02903-19, see Figure 7). Endogenous expression of ZAP is higher in monocyte-derived macrophages and THP-1 cells than CD4+ T cells. She showed that ectopic expression of ZAP in HEK293T cells is higher than these cells, though we have used a titration of ZAP to compensate for this and show a dose-dependent effect. This approach is similar to previous studies of antiviral proteins using HEK293T transfection systems such as APOBEC3 proteins and tetherin. Importantly, ZAP-L is less well expressed than ZAP-S, showing that ZAP antiviral activity does not correlate with expression levels. Indeed, we have shown the expression of all of our ZAP mutant proteins and all of the meaningful loss of function mutations have either similar or higher expression than wild type ZAP-L. Therefore, for this functional analysis, differences in expression level are not responsible for the differences in antiviral activity.

**2. Only one HIV-1 strain (WT NL4-3), a CpG-high HIV-1 mutant derived from WT NL4-3, and one SARS-CoV-2 strain (England 2) were tested. It would be helpful to test additional viral strains or a mutant SARS-CoV-2 containing more CpG.**

We appreciate this criticism by the reviewer, although it should be noted that the CpG enriched NL4.3 is an artificial viral system that allows the sensitization of a resistant virus to ZAP. Since the rules that govern context dependency of CpGs in RNA viral genomes is not really understood, we feel recoding and characterizing other HIV-1 isolates is beyond the scope of this paper. We and others have inserted CpGs into other HIV-1 isolates and found that they often inhibit in ZAP-independent mechanisms, highlighting the knowledge gaps for how ZAP targets specific viral transcripts (e.g Kmiec et al 2020, Ficarelli et al 2020, unpublished data). Furthermore, at the moment it isn’t possible practically to make wholesale changes to the CpG content of SARS CoV-2 as the reverse genetic systems are quite challenging. However, we have examined the contribution of ZAP to the IFN-gamma antiviral phenotype for both the Wuhan-like England/02 strain vs the B.1.1.7/Alpha strain. Nchioua et al 2020 previously showed ZAP contributed to the sensitivity of the early pandemic SARS-CoV-2 isolate to IFNγ, and in our A549/ACE2 ZAP CRISPR cells we can reproduce this effect for Eng02, and IFNg upregulates both isoforms (New Fig 6A and B). While there has been some debate in the literature that SARS CoV-2 is losing CpGs over the course of the pandemic, these do not appear to become fixed in the SARS-CoV-2 lineages and the lineage defining mutations in the variant of concern B.1.1.7 reduces the CpG abundance by only two CpGs (with >400 CpGs in a SARS-CoV-2 genome). B.1.1.7 is similarly IFNγ sensitive, and proportionally better rescued in ZAP KO cells, suggesting that it is at least as sensitive, if not more so, to ZAP in these assays. Thus Eng02 is not an outlier in terms of ZAP sensitivity. Furthermore, while Y108A is substantially reduced for SARS CoV-2 restriction, it still has residual activity, which may suggest CpGs are not the only ZAP target in SARS COV-2.

Whilst revising this manuscript, we found an issue with our normalization of SARS CoV-2 in culture supernatants that has impacted the data in Figure 6. We find that the ZAP-S isoform does indeed have antiviral activity against SARS CoV-2. Despite this, the CaaX-mutant of ZAP-L still loses all its antiviral activity. This data reanalysis now completely matches that N protein expression levels. Thus ZAP-S may have a distinct antiviral activity against SARS CoV-2. We have revised the text accordingly.

**Reviewer #2:**

**1. It is not easy to tell what is new here and what is confirmation of other studies either with different viruses or in different cells. The authors make a point that some previous studies could have been flawed because they used over-expression in cells that also express endogenous ZAP. However, I could not tell from the manuscript whether or not that really made a difference. For nearly every figure, the authors need to be much more clear about what are new insights here versus what are validation using a better experimental design.**

Thank you for pointing this out. We have revised the text of the manuscript to be clear what is new and what is a validation of previous experiments. Importantly, much of the ZAP functional analysis has been done using Sindbis virus and it was unclear whether some aspects of ZAP antiviral activity were specific for viruses that have a similar site and replication strategy of this virus or if they are general functional principles for the ZAP antiviral system for diverse viruses. Sindbis virus replicates in cytopathic vesicles derived from modified endosomes or the plasma membrane. Therefore, we have used HIV-1, which transcribes its RNA in the nucleus and assembles at the plasma membrane as well as SARS-CoV-2, which replicates in double membrane vesicles derived from the ER. Importantly, no previous functional analysis of ZAP had been done previously for SARS-CoV-2. Our most important finding is that targeting ZAP-L to the cytoplasmic endomembrane system is essential for full antiviral activity against both viruses, which is similar to Sindbis virus. As we now discuss, since HIV-1 does not replicate on intracellular membranes similar to positive strand RNA viruses without a DNA intermediate, this implies that ZAP is targeted to these membranes not to allow it to sense the viral RNA but instead appears to be required for proper assembly of the antiviral complex between ZAP and its cofactors. **2. Related to comment #1, I think the authors could better present what is new here by cutting down the figures and focusing the manuscript better. For example, is Figure 1 needed? Could Figure 2 be pared down and the more important parts moved to Figure 3? Do you really need some of the supplemental figures that are barely mentioned at all in the main text? I think a more concise and focused paper will help the authors in the long run. (I am not insistent that the material needs to be cut, but please consider the suggestion).**

We would like to keep Figure 1 because it addresses results previously published by other groups. First, as we discuss in the manuscript, several reports have shown that human or rodent ZAP constructs expressing the N-terminal domain consisting of the RBD plus some residues C-terminal of this domain (approximately 1-256) have antiviral activity when ectopically expressed in cells expressing wild type ZAP. However, we were not able to reproduce this data in human cells with human ZAP in the context of CpG-enriched HIV-1 or SARS-CoV-2 in ZAP knockout cells. This is important for the central hypothesis of our manuscript that ZAP localisation to the cytoplasmic endomembrane system is required for full antiviral activity against viruses with diverse replication strategies, including CpG-enriched HIV-1 and SARS-CoV-2. Second, in Meagher et al 2019 (PNAS 116:24303-24309), ZAP constructs with mutations in CpG contact residues such as Y108A and F144A were reported to partially decrease antiviral activity against CpG-enriched HIV-1 and increase ZAP activity against wild type virus in ZAP-deficient 293T cell, indicating that they abrogated CpG specificity instead of just being a loss of function mutant for antiviral activity. In contrast, Luo et al. 2020 (Cell Reports 30: 46–52) reported that Y108A had no antiviral activity against a modified Sindbis virus (SINV-nSP3-NanoLuc) in Rat2 cell expressing the ZAP in the context of NZAP-Zeo-myc. Therefore, since there was a discrepancy in terms of whether mutating CpG contact residues inhibited CpG specificity or full antiviral activity, we re-evaluated the role of the RBD for wild type and CpG-enriched HIV-1 in Figure 1. We do not know why our results were different from Meagher et al 2019, but in those studies it appears that they used ZAP and TRIM25 knockout cells in which both ZAP and TRIM25 were ectopically expressed, which may contribute to the differences observed. Overall, we believe that that Figure 1 is useful because it addresses previously published results that do not form a coherent model for ZAP antiviral activity.

We do agree with the reviewer that the paper was perhaps too data dense at the start. Thus, we have decided to move what was Figure 4 about PARP12 (which essentially backs up Fig2 and 3). This has now been incorporated into Figure S3 to streamline the figures.

**3. Figure 6: The increased amount of pull-down of TRIM25 and KHNYN by ZAP-L seems over-interpreted. The amount of increased pull-down may be statistically significant, but does not really seem to explain the extent of increased activity of ZAP-L relative to ZAP-S, and the ZAP-S+ CVIS does not attain the amount of binding of ZAP-L. It seems to me that there is more going on here. Finally, please work on the figure legend to make it easier to the data presented in the figure.**

We agree that we do not have a full understanding of what the ZAP antiviral complex is composed of and how it is assembled. Mutating the CaaX box leads to a moderate decrease in the interaction with KHNYN and TRIM25, indicating that other regions in ZAP may also regulate this interaction. Importantly, adding the CVIS to ZAP-S also moderately increases the interaction between ZAP-S and KHNYN, indicating that localisation to the cytoplasmic endomembrane system partially regulates this interaction. While the primary KHNYN binding region is are localized to the N-terminal domain of ZAP (1-352) by yeast-2-hybrid experiments, this is not sufficient to efficiently co-immunoprecipitate KHNYN, even when the CaaX box is added to this construct, indicating that assembly of the antiviral complex in a human cell requires other domains in ZAP. We have clarified this in the text and rewritten the legend for Figure 6 to present the data more clearly.

**Reviewer #3: I see no need for major new experiments.**

**Part III – Minor Issues: Editorial and Data Presentation Modifications
Please use this section for editorial suggestions as well as relatively minor modifications of existing data that would enhance clarity.**

**Reviewer #1:**

**1. Fig. 1B legend: HIV-1 in the ZAP mutant should be HIV-1 WT. AUC plots (Fig. 1B, 1D, 2B, 2E and others) can be labeled better with virus strains used. Fig. 1D right panel: WB data show F144A significantly reduce Env expression in cells and virions. This point should be discussed.**

Thank you for pointing ways to improve the labelling for Figure 1, we have fixed this to make it more clear. We agree that ZAP-L F144A does reduce Env expression for the CpG-enriched HIV-1, though not to the level observed for wild type ZAP-L, and have highlighted that this is not a complete loss of function mutant in the text.

**2. Line 164: It seems that the sentence should read: “However, this does NOT fully account for the loss of phenotype…”**

Thank you, we have fixed this sentence. **3. Line 197, cite figure number in the sentence.**

Thank you, we have now cited the figure number. **4. Line 223: insoluble fractions (should be I not D) as showed in Fig. 5.**

Thank you, we have fixed this. **5. Fig. 6: A and B panel labels are missing.**

Thank you for pointing this out. We have decided not to divide the figure into panels A and B and have fixed the text accordingly.  **6. Fig. 7: It would be more convincing if the authors show dose-dependent inhibition of SARS-CoV-2 by ZAP-L as they did for HIV-1 infection assays. Moreover, it would be helpful to show other SARS-CoV-2 protein expression in addition to the N protein.**

While we have added data to the new figure 6, our re-analysis of supernatant genome data now mirrors the N expression in the cells much better. As N is the best expressed protein in the virus life-cycle, in our hands it is the most robust for these assays. Furthermore, Nchioua et al has shown that ZAP has comparable effects on N and S subgenomic RNA. They have also shown that both ZAP-L and ZAP-S inhibit SARS-CoV-2 in a dose-dependent manner, with ZAP-L being the more active isoform, which is supported by our data. However, the functional motifs required for ZAP-L antiviral activity had not been previously characterized, which is the novel insight for this figure.

**Reviewer #2:**

**4. Line 80: The role of CG-richness is less clear for other viruses (e.g. for the alphaviruses or for MLV) than it is for the CG-rich HIV-1 targets used here and by others. The authors should qualify this statement to clarify what has been tested versus what is hypothesis.**

Thank you. We have rewritten this sentence to make it more clear what has specifically been hypothesized and what has been experimentally determined. **5. Line 86: TRIM25 was first found to be a co-factor for ZAP in the alphavirus system and then for MLV, not the CG-rich HIV-1 system (**[**https://journals.plos.org/plospathogens/article?id=10.1371/journal.ppat.1006145**](https://journals.plos.org/plospathogens/article?id=10.1371/journal.ppat.1006145) **and** [**https://pubmed.ncbi.nlm.nih.gov/28202764/**](https://pubmed.ncbi.nlm.nih.gov/28202764/)**)**

Thank you, we have clarified this by rewriting this sentence as well as other paragraphs in the introduction.

**Reviewer #3: Specific issues:
The data in Fig. 1B and C really look different from the way they are presented in the text. While the ZAP WT control always looks nice, all the mutants frankly look inactive on any substrate. There’s no change with increasing ZAP. I don’t see that Y108A has any activity on either target – no different than the ∆RBD.
Fig 1D has bars for “ZAP-L”, but where is that data – is that the same as ZAP WT CTRL in B and C?**

We apologise for the confusion in Figure 1. We have redone it to clarify the samples that are being analysed.

**ll. 116 ff: A very confusing sentence. “Co-transfection of full-length ZAP….” with virus gave a modest inhibition of both normal HIV-1 and CpG-high HIV-1, but “wild type ZAP” gave potent inhibition of CpG-high HIV-1. What’s the difference between full-length ZAP and wild type ZAP??**

We agree that this was confusing and have rewritten the sentence.

**ll. 124 ff: The description here seems unnecessarily complex: “the phenotype of RBD deletion could be phenocopied by five alanine substitutions in the proposed RNA binding groove”. Couldn’t one just say something like “A mutant with five alanine substitutions in the proposed RNA binding groove also had no activity”?**

Thank you, we have clarified this by revising the text.
 **ll. 132: Statements like “the RBD of ZAP is essential yet insufficient for CpG-mediated restriction of HIV-1” may be true but doesn’t say if the RBD is sufficient for some activity – for some kind of non-CpG-mediated activity. What exactly do we mean by CpG-mediated activity vs. non-CpG-mediated activity? Some kind of basal activity?**

Thank you, we have rewritten this sentence to make it clearer.

**ll. 137: Confusing: “deletion of the PARP domain resulted in an almost complete loss of CpG-specific inhibition”. So do we take it that the deletion had no effect on the activity against a CpG-low target? If so, we need to hear that. Maybe we need to see the high/low ratio, or levels of both activities.**

Thank you, we now note that it had no activity against either virus.
 **line 152: I guess we know these triad motif mutations (in other settings) block PARP activity, but why do we think they disrupt “the structural integrity of the ZAP PARP domain”? I would have thought they were chosen not to disrupt overall structure.**

Thank you, we agree that this was a confusing statement and have rewritten it to make it clearer. The structural integrity of the PARP domain was not specifically analysed, the important point concerns the residues that make up the NAD+ binding site in active PARP domains.

**While it seemed like a good idea that ZAP-S might only be working in concert with endogenous ZAP-L, the experiment to directly test this (line 174) seemed to eliminate this.**

We agree with the reviewer. Because it was a formal possibility, we did the experiment in Fig S2 to directly test this.

**The PARP12/ZAP chimeras seem confusing. Not sure what is concluded from them. Are they helpful?**

ZAP evolved from a PARP12 duplication during tetrapod evolution and we find it interesting to determine how these proteins have evolved independently to have different antiviral activity. Since PARP12 has no antiviral activity against wild type or CpG-enriched HIV-1, it also acts as a useful tool for gain of function experiments to identify the essential domains in ZAP required for CpG-specific restriction. We found that there are functional differences both in the RBD and C-terminus of the two proteins and transferring both the ZAP RBD and CaaX box is required for PARP12 to have partial antiviral activity on CpG-enriched HIV-1. This highlights how these motifs are required for ZAP activity using a gain-of-function phenotype. However, to help streamline the main manuscript, we have removed the main figure and incorporated this data into Fig S3.

**Line 219: What’s the conclusion of the poly(I:C) experiment? Presumably this is inducing stress granules or other ISGs, but why are the effects on ZAP localization not requiring the CaaX box? I didn’t understand how these results are interpreted.**

Previous studies have concluded that ZAP association with stress granules is important for antiviral activity against Sindbis virus. Transfection of poly (I:C), which induces stress granules through activation of PKR, reveals, as expected, that ZAP-L and ZAP-S localise to stress granules. However, both isoforms do so similarly and, importantly, mutation of the ZAP-L CaaX box has no effect on stress granule localisation despite a complete loss of antiviral activity. Thus ZAP-L viral restriction and stress granule localization do not correlate for CpG-enriched HIV-1 or SARS-CoV-2. We believe this suggests they are not required for the antiviral activity of ZAP-L, at least against the viruses tested here. We have rewritten this section to make it clearer. **Line 283: It wasn’t until this point very late in the paper that I was able to figure out that in this experimental system “ZAP-S has no antiviral activity on its own”. Maybe this could have been said earlier, along with the fact that it has activity in other settings. That alone would seem to be an important and interesting point to make, justifying some speculation as to the differences between the various assays.**

Thank you for highlighting this point. We have rewritten the section for Figure 3 to make this clear.

**Line 348: I don’t understand the logic of the sentence “the evolution of CpG-specific antiviral activity enhanced by the PARP domain in ZAP led to, or was a consequence of, the loss of its own ADP ribosylation ability.” Needs more explanation.**

We agree and this sentence has now been deleted as we have rewritten the discussion to address all of the reviewer’s comments.
